# Quantification of motor network dynamics in Parkinson’s disease by means of landscape and flux theory

**DOI:** 10.1371/journal.pone.0174364

**Published:** 2017-03-28

**Authors:** Han Yan, Jin Wang

**Affiliations:** 1 State Key Laboratory of Electroanalytical Chemistry, Changchun Institute of Applied Chemistry, Chinese Academy of Sciences, Changchun, Jilin, P.R.China; 2 Department of Chemistry and Physics, State University of New York at Stony Brook, Stony Brook, New York, United States of America; Florey Institute of Neuroscience and Mental Health, The University of Melbourne, AUSTRALIA

## Abstract

The basal ganglia neural circuit plays an important role in motor control. Despite the significant efforts, the understanding of the principles and underlying mechanisms of this modulatory circuit and the emergence of abnormal synchronized oscillations in movement disorders is still challenging. Dopamine loss has been proved to be responsible for Parkinson’s disease. We quantitatively described the dynamics of the basal ganglia-thalamo-cortical circuit in Parkinson’s disease in terms of the emergence of both abnormal firing rates and firing patterns in the circuit. We developed a potential landscape and flux framework for exploring the modulatory circuit. The driving force of the circuit can be decomposed into a gradient of the potential, which is associated with the steady-state probability distributions, and the curl probability flux term. We uncovered the underlying potential landscape as a Mexican hat-shape closed ring valley where abnormal oscillations emerge due to dopamine depletion. We quantified the global stability of the network through the topography of the landscape in terms of the barrier height, which is defined as the potential difference between the maximum potential inside the ring and the minimum potential along the ring. Both a higher barrier and a larger flux originated from detailed balance breaking result in more stable oscillations. Meanwhile, more energy is consumed to support the increasing flux. Global sensitivity analysis on the landscape topography and flux indicates how changes in underlying neural network regulatory wirings and external inputs influence the dynamics of the system. We validated two of the main hypotheses(direct inhibition hypothesis and output activation hypothesis) on the therapeutic mechanism of deep brain stimulation (DBS). We found GPe appears to be another effective stimulated target for DBS besides GPi and STN. Our approach provides a general way to quantitatively explore neural networks and may help for uncovering more efficacious therapies for movement disorders.

## Introduction

The neural network is a complex dynamical system [1–4]. Individual neurons connected with each other perpetually generate complex patterns of activities that are responsible for specific cognitive functions. Some brain regions are characterized due to their activities being directly correlated with the specific cognitive behaviors. For example, the activity of decision-associated brain areas, as that of lateral intraparietal (LIP) neurons, is correlated with the response times and choices in decision-making tasks [5, 6]. While, on the other hand, some brain regions play a significant role in physiological process by modulating the functions of other regions. The basal ganglia neural circuit, as a typical example of the latter kind of brain region, is well-known due to its crucial role in movement control [7]

In the past decades, great efforts have been made in understanding the basal ganglia neural circuit, since the Parkinson’s disease(PD) is closely related to the basal ganglia dysfunctions [8–11]. Classical models have described PD symptoms in terms of altered firing rates along the direct/indirect pathways in basal ganglia [12]. Dopamine depletion leads to decreased activity over the direct pathway and increased activity over the indirect pathway. Both of these changes result in the increase of average firing rate of the basal ganglia output nuclei GPi/SNr. This induces over-inhibition of thalamic and cortical activity from GPi/SNr, thereby suppressing movements. Although many evidences support the hypotheses that PD results from the changes in firing rate and imbalance between the direct and indirect pathways in the basal ganglia [9, 13, 14], this hypothesis was challenged by the recent electrophysiological studies that fail to show the expected significant changes of firing rates in the pallidum, thalamus or motor cortical areas of MPTP monkeys [13, 15]. This shifted the attention of researchers to the abnormal firing patterns such as oscillations and neuronal synchronization in the pathophysiology of PD rather than the changes in the firing rate, since an increased tendency of basal ganglia neurons to fire in an oscillatory manner has also been identified in PD [13, 15, 16]. In addition, the original firing rate description cannot easily explain tremor in the PD, which is another obvious cardinal feature of PD. The mechanism of tremor is still in debate. We can see some hints from recent experimental evidences showing that when tremor is present, an increased tendency of basal ganglia neurons to fire in an oscillatory manner has also been identified in Parkinsonism [15–21].

In order to uncover the origin of abnormal neuronal oscillations in the basal ganglia circuit that may contribute to parkinsonian symptoms, some computational studies used the inhibitory-excitatory loop architecture of the STN-GPe network for the generation of oscillatory behavior in PD [22–24]. However, there are evidences showing that an isolated subthalamic-globus pallidus circuit in vitro is not sufficient to generate pathological oscillations [25]. In addition, synchronized oscillatory activity not only appears in STN-GPe but also closely links to oscillations in SNr and cortex in PD [26, 27]. Therefore, inputs from other regions, such as the cortex, are also necessary for generating pathological oscillatory activity. Furthermore, the deep brain stimulation as a clinically therapy for PD has been shown to be effective at more than one region of the basal ganglia-thalamo-cortical circuit [28, 29]. These findings suggest that the whole basal ganglia-thalamo-cortical circuit may be associated with synchronized oscillatory activity in PD. Therefore, in this work we developed a network model to capture the core components in the basal ganglia-thalamo-cortical circuit, including the major connections between these neural populations.

The realistic neural networks are influenced by intrinsic and extrinsic fluctuations. Therefore, the global stability of the motor network in pathological condition, namely the robustness of the oscillatory activity in the whole basal ganglia-thalamo-cortical circuit still needs to be quantified. However, the global dynamical natures of this system are hard to explore by just following the individual population trajectories in finite short times. We meet the challenge by applying the landscape and flux theory to the dynamical neural network [30–36]. For an equilibrium system, the potential landscape of the system is related to the equilibrium probability distributions based on the Boltzman law. The driving force of the system can be written as a gradient of the potential function. Since there are always constant exchanges of materials or energies with the outside environment, neural circuits should be considered as non-equilibrium systems rather than equilibrium systems. Analogous to the potential landscape in equilibrium systems, we can still construct the potential function of a dynamical neural system associated with corresponding steady state probability distributions. However, the driving force of the non-equilibrium neural system cannot be written purely as a gradient of the potential function. We found the driving force of non-equilibrium neural networks with explicit detailed balance breaking can be decomposed into two components: the gradient of the potential and the curl probability flux. Such probability flux represents probability flow in state space of the activity of the neural circuit. In the landscape framework, certain significant changes of physiological behavior can be understood as the transition from an attractor state to another [4, 37]. Different from the energy landscape in the original Hopfield neural models, the quantification of potential landscape in our study can be applied to general neural networks without the restriction on the symmetrical connections [4]. The probability flux provides an important driving force for the large-scale oscillations in the neural circuit, such as abnormal oscillations in movement disorders.

Dopamine is a key neuromodulator in the basal ganglia-thalamo-cortical circuit [38]. We quantified the probabilistic landscapes of the neural circuit for different dopamine levels. With sufficient dopamine supply in the normal state, the landscape topography shows mono-stable basin of attraction and there is no significant synchronized oscillatory behavior. When there is not enough dopamine in the basal ganglia, we show that the system goes through a Hopf bifurcation to beta-band oscillations. The shape of the quantified landscape changes from single basin of attraction to a Mexican hat-shape closed ring, which corresponds to the abnormal oscillations in PD. We found both landscape and flux contribute to the dynamics of oscillations. The landscape attracts the network state down to the ring, and the curl flux drives the oscillations along the ring path. We quantified the global stability through the barrier height of the center hat. Both higher barriers and larger flux result in more stable oscillations. We found that energy consumption is needed for supporting the stronger synchronized oscillations. We also explore the effects of dopamine level on the frequency of oscillations. Moreover, by means of the landscape and flux approach, we provided theoretical supports for the therapeutic mechanisms of the deep brain stimulation in terms of the effective reduction of the synchronized oscillations in the circuit. According to our theoretical analysis, GPe appears to be another effective target for DBS besides GPi and STN. Our theoretical predictions may be helpful to uncover more efficacious therapies for movement disorders, such as the Parkinson’s disease.

## Results and discussion

### The landscape and flux theory for general neural networks

When exploring the dynamics of a neural network, individual deterministic trajectories are often followed. However, the neural networks are under fluctuations from the intrinsic source and external environments. In addition, following individual trajectories in finite short times often gives local properties rather than global natures of the whole system. Rather than individual trajectory evolution, the probabilistic evolution can characterize the dynamics globally and therefore is often more appropriate. In a recent work, we applied the landscape and flux theory for studying general neural networks [34]. We quantified the potential landscape related to the steady state probability distribution. This is analogous to equilibrium systems where the potential landscape is related to the equilibrium distribution through the Boltzman law. Here the potential function becomes *U*(**x**) = −*ln*(*P*_*ss*_(**x**)), where *P*_*ss*_ represents the steady state probability distribution and **x** is a vector variable representing the activity of each module in the circuit.

To quantify the steady state probability distribution, we can start with the dynamical equations of the neural network taking the fluctuations into account. A set of Langevin equations describing the stochastic dynamics of neural networks can be written as: $\frac{dx}{dt}=F\left(x\right)+\zeta$. Here **F** represents the driving force of the neural network. *ζ* represents the stochastic fluctuations, which are considered to follow a Gaussian distribution with autocorrelations specified by $<\zeta \left(x,t\right)\zeta \left(x,t^{\prime }\right)>=2D\left(x\right)\delta \left(t-t^{\prime }\right)\;$. Here **D**(**x**) is the diffusion coefficient matrix characterizing the level of noise strength. *δ*(*t*) is a delta function. Furthermore, we can write the corresponding diffusion equation in the form of the probability conservation: ∂*P*/∂*t* + ∇ ⋅ **J** = 0(see more details about solving the probability distributions in the Method section), where **J** is the probability flux: **J** = **F***P* − ∇ ⋅ (**D***P*). Here the diffusion coefficient matrix is considered as a constant for simplification. For a general equilibrium system, one often knows the energy function a priori and the driving force is the gradient of the energy function. However in reality, the neural networks are open systems. The neural circuits often exchange energy and information with the environment, which cannot be taken as an isolated conserved systems. The driving force as a result can not always be written as a gradient of an energy function in such non-equilibrium conditions. According to the expression of the probability flux **J**, we can decompose the driving force of the neural systems into a gradient of a non-equilibrium potential and a divergent free curl flux force [30–32] as: **F** = **J**_*ss*_/*P*_*ss*_ − **D** ⋅ ∇*U*. The divergent free flux **J**_*ss*_ has to rotate around and becomes curl since it has no source or sink to go to or come out from. When the steady state flux is zero, there is no net flux flow in or out; the system is in a detailed balance. When the flux is not zero, there is a net steady state flux flowing around. This breaks the detailed balance and measures how far away the system is from the equilibrium. Therefore while the nonequilibrium potential quantifies the landscape and reflects the probability of each state giving a global characterization, the dynamics of neural networks is determined by both the gradient of the landscape and the curl flux breaking the detailed balance.

### Application of landscape and flux theory for the quantification of motor network dynamics

**Fig 1:**
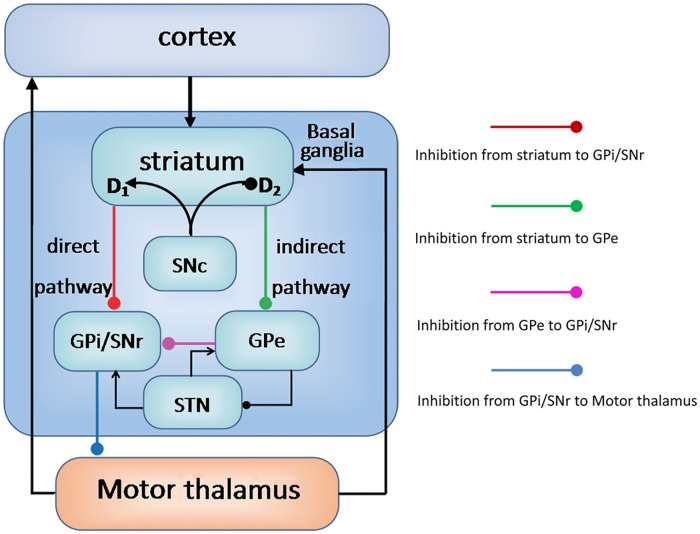
The schematic diagram of the basal ganglia-thalamocortical circuity and their interactions. Arrows represent excitatory connections and the lines with solid circle represent inhibitory connections.

In this work, we constructed a neural network model which includes most of the main modulation modules in the basal ganglia-thalamo-cortical circuit [8–11, 39–41] as shown in Fig 1. The basal ganglia is closely connected with the cortex and thalamus. Excitatory inputs from the cortex and thalamus enter the basal ganglia through the striatum, where they are transformed into two inhibitory projections called the direct and indirect pathways. In the direct pathway, striatal neurons form inhibitory synapses to the GPi/SNr complex. The GPi/SNr complex serves as the output nuclei and connects to the cortex via the motor thalamus. On the other hand, the indirect pathway striatal neurons provide inhibition to the GPe, which in turn inhibits the SNr/GPi. The circuit forms a closed loop due to the inhibition from SNr/GPi to the thalamus. The opposite effects of these two pathways control the movements. Dopamine level dynamically modulates this circuit. It activates the direct pathway and inhibits the indirect pathway through the D1 and D2 receptors in the striatum, respectively. The pathophysiology of PD has been considered to be caused by the lack of balance between the excitatory and inhibitory control. In the classical model, the Parkinson’s disease was understood as the results from dopamine depletion in the SNc, which leads to decreased activity over the direct pathway and increased inhibitory activity over the indirect pathway [9, 10, 13]. In addition, there is another hyperdirect pathway, which goes from the cortex to the subthalamic nucleus(STN)and then to the GPi in the loop. Activation of this pathway would also increase basal ganglia output and result in greater inhibition of thalamocortical activities.

**Fig 2:**
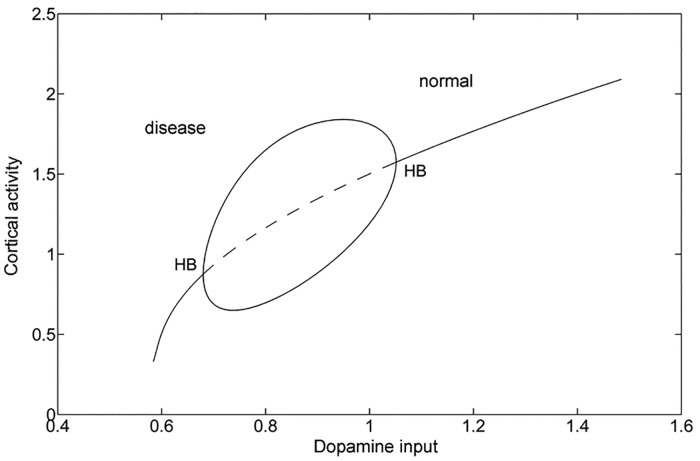
The phase diagram of the basal ganglia-thalamocortical circuit in terms of varying dopamine input. The solid line indicates the mono-stable phase and the dashed line indicates the unstable oscillation phase. HB represents the Hopf bifurcation point. (A Hopf bifurcation is a critical point where a dynamical system loses stability and switches from a fixed point to a periodic solution.)

Based on the neural network model shown in Fig 1, the dynamics of the system can be quantitatively described by a set of ordinary differential equations as $\frac{dx}{dt}=F\left(x\right)$. In this work, each module is represented by a single Hopfield-type model neuron. Similar simplification is also used in previous computational studies [24, 42]. In Fig 2, we show the phase diagram with changing dopamine input derived by the analysis of the deterministic equations for neural network dynamics. We found that the average activity of the motor cortex decreases gradually with decreasing dopamine. This corresponds to the descriptions in the classical firing rate model that dopamine depletion leads to inhibited thalamocortical activity resulting in akinesia [9, 10, 13]. Moreover, as the dopamine supply decreases, system goes through a Hopf bifurcation from a mono-stable phase to a limit cycle oscillation phase.

**Fig 3:**
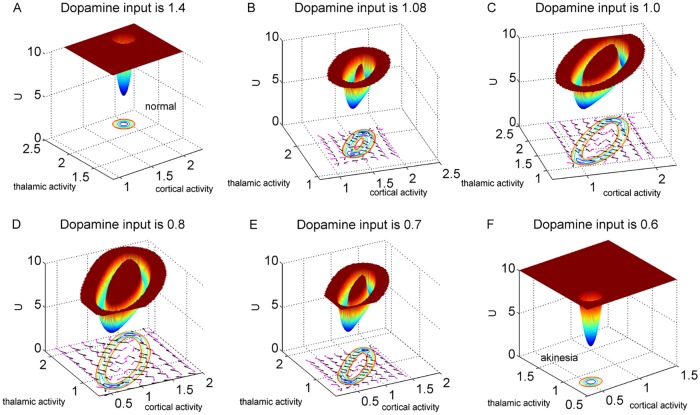
Potential landscapes based on a 2-dimensional state space for different dopamine levels. The dopamine input is 1.4, 1.08, 1.0, 0.8, 0.7, 0.6, respectively. The black arrows represent the negative gradient of potential and pink arrows represent probabilistic flux.

Given the equations that can describe the dynamics of the neural circuit, we can quantify the corresponding potential landscapes in the state space. Since the cortex and thalamus directly determine the movement execution, we focus on 2-dimensional state space (instead of a 7-dimensional state space describing the activity of the 7 components in the circuit) for better visualisation of potential landscape. To address whether the results are also valid for the other nuclei activities, we show corresponding results in the state space of GPi and STN activity in the S1–S4 Figs. In fact, choosing any two nuclei activities for the 2-dimensional state space does not significantly change the theoretical predictions we get. The landscapes of the basal ganglia-thalamo-cortical circuit with different dopamine levels in terms of motor cortex and thalamus module activities are shown in Fig 3. In Fig 3(A)–3(F) we showed three-dimensional landscapes for decreasing dopamine inputs. The states with lower potentials correspond to higher probabilities. We see in Fig 3(A), with sufficient dopamine release, there is a basin of attraction that corresponds to the state with both high activities of motor cortex and thalamus. This is due to the increased activity over the direct pathway and decreased activity over the indirect pathway. Once stimulated, the cortex projects its own excitatory outputs to the brain stem and ultimately muscle fibers, thus enabling the movement execution. This attractor can be regarded as the normal state. Dopamine depletion leads to less excited direct pathway and over-activated indirect pathway. Therefore, the enhanced outputs from the GPi over-inhibit the thalamocortical projection, and furthermore reduce the cortical neuronal activation associated with the movement initiation. As shown in Fig 3(F), when the dopamine release is extremely low the basin of attraction is located at the state where both motor cortex and thalamus have lower activities. These results are consistent with the descriptions in the classical firing rate model [9, 10, 13].

As we discussed in the introduction section, dopamine loss promotes the tendency of neurons in the basal ganglia-thalamo-cortical circuitry to generate oscillatory firing patterns. In our model, when the dopamine supply decreases, we found the landscape of the circuit changes from a single basin of attraction to oscillations with a Mexican hat shape as shown in Fig 3(B)–3(E). In Fig 3(B)–3(E), we can see the oscillation path along the ring valley has lower potentials or higher probabilities. One should also notice that the potentials along the ring are inhomogeneous. There are two local basins on the oscillation ring. The two basins correspond to the state with both higher cortical and thalamic activities as well as the state with both lower activities. The inhomogeneous potentials along the oscillation ring imply the system may converge to these basins of attraction when the oscillation becomes unstable. This can be seen that at high dopamine release level, the network settles at the normal state while for extremely low release of dopamine, the network settles to the disease state, where the neural circuit is not able to execute movement(akinesia). The stabilities of oscillations are discussed in the next section. In Fig 3(B)–3(E), the pink arrows represent the probabilistic flux, and the black arrows represent the force from negative gradient of the potential landscape. We can see the direction of the flux near the ring is parallel to the oscillation path. The direction of the negative gradient of the potential is almost perpendicular to the ring. Therefore, the landscape attracts the system towards the oscillation ring, and the flux is the driving force and responsible for coherent oscillation motion on the ring valley.

### The effects of dopamine on the motor circuit in terms of barrier height, flux, period and energy consumption

**Fig 4:**
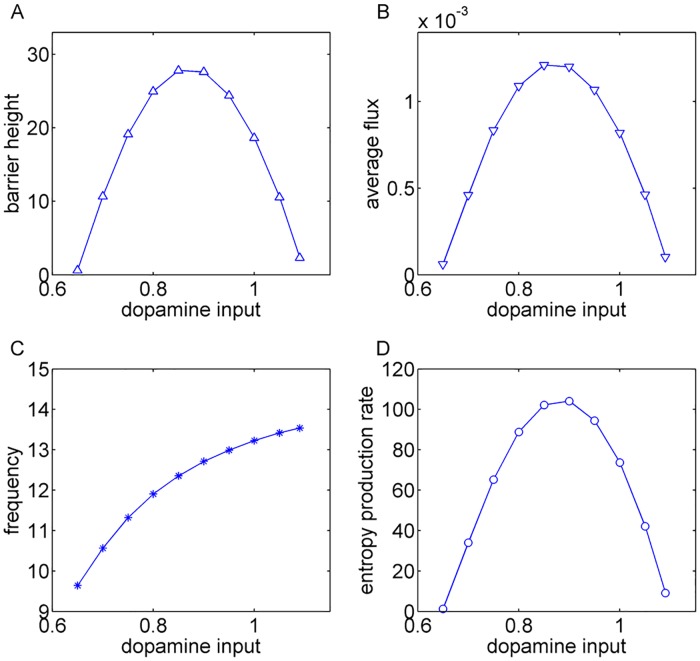
The effects of dopamine on the motor circuit in terms of barrier height, flux, period and entropy production rate. (A) and (B) show the changes in barrier height and flux with varying dopamine input. With enough dopamine supply, there are no oscillations. Oscillations may emerge as the dopamine input decreases. However, the oscillations are not stable when the dopamine input is around the Hopf bifurcation point. The corresponding barrier heights and flux are smaller. The oscillations become more stable as the dopamine input decreases further. Accordingly, the barrier heights and flux increase. However, when the dopamine supply is extremely low, both motor cortex and thalamus are suppressed. The oscillations become unstable again. The corresponding barriers and flux also decrease. (C) shows the period of oscillations decreases monotonously with increasing dopamine input. More dopamine results in higher frequencies for oscillations. (D) shows the entropy production rate increases for more stable oscillations. The range of dopamine input is limited for generating oscillations.

Since the emergence of pathological oscillations has been demonstrated in many movement disorders, especially the Parkinson’s disease [15–17, 43], exploring the mechanism and stability of such oscillations is crucial for not only the tremors but also other symptoms such as akinesia. Here we studied how the dopamine, as the key modulatory factor, influences the oscillatory dynamics in motor circuits. Having quantified the potential landscape, we can further study the robustness and global stability of oscillatory activity and the functions of the motor network in PD by exploring the landscape topography. We used the barrier height as a quantitative measure which is defined as *U*_*max*_ − *U*_*min*_. *U*_*max*_ is the local maximum potential inside the cycle ring, and *U*_*min*_ is the minimum potential along the ring. For higher barriers, it is more difficult to go from the oscillation path to the inside or outside of the oscillation ring valley. Therefore, the system is more likely to move along the ring step by step rather than jumping from one location of the oscillation ring to another directly by crossing the barrier. This means higher barrier height leads to more robust and coherent oscillations. In Fig 4(A), as the dopamine release increases, the barrier height increases at first then decreases. This result is consistent with the potential landscapes shown in Fig 3. With enough dopamine supply, the potential landscape of the system is a basin of attraction and there are no oscillations. Oscillations may emerge as the dopamine input decreases. However, the oscillations are not stable when the dopamine input is around the Hopf bifurcation point. The corresponding barrier heights are lower. The abnormal oscillations become more stable as the dopamine input decreases further. Accordingly, the barrier heights become higher. However, when the dopamine supply is extremely low, both motor cortex and thalamus are suppressed. The oscillations become unstable again. The corresponding barriers also become lower.

We have shown that the flux is the main driving force of the non-equilibrium system after being attracted into the closed oscillation ring. Therefore, the flux is another good measure to quantify the stability of oscillations, since the larger flux means that the coherent oscillations are harder to be influenced by the fluctuations around. Here we use the average flux along the cycle to quantify the magnitude of flux, which is defined as $J_{Average}=\frac{\oint Jdl}{\oint dl}$. We can see in Fig 4(B) that we got similar results as shown in Fig 4(A). The average flux increases first then decreases with increasing dopamine due to emergence and disappearance of oscillations. In conclusion, both the barrier height and flux are crucial for the synchronized oscillations observed in motor disorders.

The frequency is a crucial feature of pathological oscillations in PD [13, 15, 16]. In Fig 4(C), we explored how the dopamine influences the frequency of oscillations. Interestingly, the trend of the oscillation period with varying dopamine level doesn’t show non-monotonic behavior. The frequency of oscillations increases monotonically with increasing dopamine level. Although the frequency increases monotonically, the oscillations in our model are still in the beta band. There are some plausible hypothesis showing that the beta band oscillations may be related to the hypokinetic symptoms as well as tremor in PD [18, 44–46]. Although the oscillations in our model are not exactly at the tremor frequency, quantitatively exploring such oscillatory behaviors in beta band is still useful for understanding the mechanisms of movement disorders such as tremor. We found the curl flux force *J*_*ss*_/*P*_*ss*_ approximately plays the role of velocity when the oscillating system moves in the state space [33, 34]. Therefore, the flux is closely related to the period of oscillations. It is natural to expect that the shorter period of oscillations are accompanied by the larger flux. Our results shown in Fig 4(B) and 4(C) are not incompatible with this prediction. This is because the oscillation period is determined by both the flux(velocity) and the loop length of the cycle. We show that the flux multiplied by the period has positive correlations with loop length(details are shown in the S1 Text). Our predictions that the frequency of beta oscillations decreases as PD develops(fewer dopamine) are consistent with previous simulation results [22].

As we introduced before, the dysfunction of the motor circuit may result from the pathological oscillations in the basal ganglia, which disrupt the regular discharges of basal ganglia output nuclei. Irregular firing in GPi cells were also observed in generalized dystonia [47]. The changes in disorder(entropy) of the neuronal activity in the circuit can be quantified through the flux in our model(the detailed definition of entropy production rate is shown in the S1 Text). We calculated the changes of entropy production rate with respect to the dopamine. The Fig 4(D) shows that the entropy production rate is larger with higher barrier height and stronger flux, which means more stable pathological oscillations correspond to larger entropy prediction. A proposed therapeutic mechanism of STN deep brain stimulation (DBS) is that the high frequency stimulation changes the firing of GPi neurons from an irregular pattern into a regular pattern(reducing the entropy) [48]. We will show more details about the mechanisms of DBS in the next section.

In addition, the entropy production rate in our model can be seen as the energy consumption or cost per unit time [33, 35, 36]. Our results predict that more energy is needed to support the large-scale oscillatory neuronal activity over the circuit with stronger oscillations. It should be noted that the energy cost here represents the consumption of energy to maintain the dynamics of the whole neural circuit, a true non-equilibrium system. Therefore, such energy consumption or cost cannot be easily measured by the recordings of the glucose utilization and **O_2_** consumption in individual neural populations [49]. However, we can still see support from some experimental results. A previous experiment shows that oscillatory synchronization is only found in the patients with limb tremor but not in the patients without tremor [46]. This finding indicates that there may be more oscillations in the tremor dominant(TD) PD than the non-tremor dominant(NTD) PD. Furthermore, there is another evidence showing reduced blood oxygenation level dependent(BOLD) activity in the NTD PD patients compared to the TD PD patients in several areas(cortex, GPi, GPe and thalamus) [50]. Taking these results together, it is logical to hypothesize that more oscillatory activity in PD requires more energy, which is consistent with our theoretical predictions.

### Global sensitivity analysis on landscape and flux for identifying the key connections in the motor circuit

**Fig 5:**
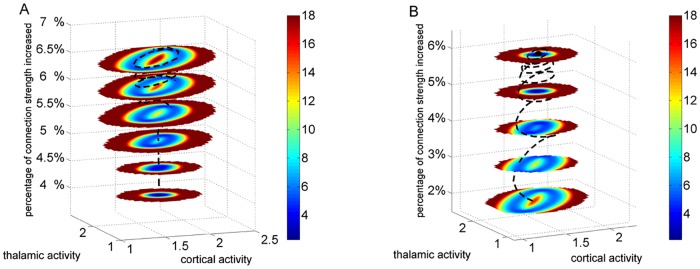
Potential landscapes of the modulation circuit for different interaction strength. (A): Increased inhibitory connection in the indirect pathway. (B): Increased inhibitory connection in the indirect pathway. As the inhibitory connection from the striatum to GPe in the indirect pathway becomes stronger the landscape changes from a basin of attraction to oscillations. On the contrary, increasing the inhibition from striatum to GPi in direct pathway results in the changing from stable oscillations to a basin of attraction. In order to show the landscapes of the system switching from the fixed point to oscillations and oscillations to the fixed point, the dopamine input is set as 1.00 and 1.25 in Fig 5(A) and (B), respectively. The black lines represent the trajectories of the system switching between the fixed point and oscillations.

Quantifying the flux and potential landscapes helps us to understand the mechanisms of how synchronized oscillations emerge in the motor circuit. We have shown that both barrier height and flux are good measures of the global stability and coherent oscillations of the system. Here we will explore the effects of some key regulation connections on the circuit. As discussed before, the output of basal ganglia is determined by the balance between the direct pathway and indirect pathway. The direct pathway promotes the movement while the indirect pathway suppresses the movement. In Fig 5(A) and 5(B), we show the potential landscapes with increased activity over the indirect pathway and direct pathway, respectively. The black lines represent the trajectories of the system switching from the fixed point to oscillations and oscillations to the fixed point, respectively. We can see that as the inhibitory connection from the striatum to GPe in the indirect pathway becomes stronger the landscape changes from a basin of attraction to oscillations. Meanwhile, the oscillations become more stable. On the contrary, increasing the inhibition from striatum to GPi in direct pathway makes the oscillation unstable. This indicates that direct-pathway activation help to alleviate the corresponding symptoms in PD. Our theoretical predictions are supported by the experimental evidences that activation of the direct pathway neurons with the optogenetic tool can ameliorate a series of motor deficits in a mouse model of PD [14].

**Fig 6:**
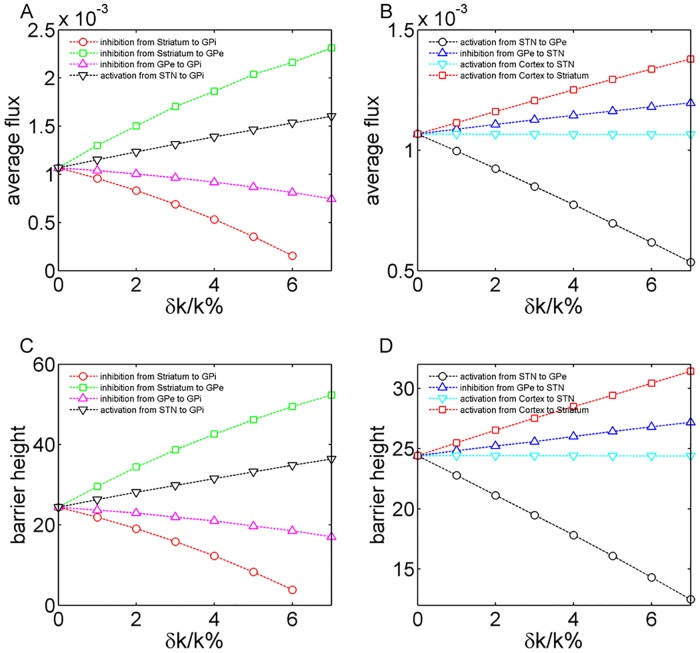
The global sensitivity analysis in terms of the barrier height and flux. Fig 6(A) and (B) show the changes in barrier heights when different wirings are strengthened. Fig 6(C) and (D) show the corresponding changes in flux. The horizontal axis represents the percentage changes in the specific connection strength. Here the dopamine input is 0.95.

Furthermore, we explored the details of how the key modulatory connections in the circuit influence the system in terms of barrier height and flux. Similar with the discussion above we enhanced different inhibitory connections separately in the circuit shown in the Fig 1. Fig 6(A) and 6(B) show the changes in barrier heights when different wirings are strengthened. Fig 6(C) and 6(D) show the corresponding changes in flux. The vertical axis represents the percentage changes in the specific connection strength. Our predictions are consistent with a recent theoretical work which shows that the connections from cortex to striatum and striatum to GPe play crucial roles in generating pathological oscillations [51].

During the last decades, the deep brain stimulation (DBS) has become an effective therapy for the treatment of numerous movement disorders [28, 29, 52]. However, the therapeutic mechanism of DBS remains unclear. The earliest hypotheses on DBS mechanisms arise from the fact that DBS has a similar effect to a lesion on parkinsonian symptoms [28, 53]. Accordingly, DBS was originally considered to inhibit neuronal activities around the stimulated sites. This hypothesis was supported by the recordings of reduced somatic activity in GPi and STN neurons during GPi and STN DBS respectively [28, 53]. In addition, the original firing rate model provides a good explanation why local inhibition of GPi or STN should be therapeutic. Therefore, the therapeutic mechanism of DBS was once explained by the direct inhibition hypothesis. However, the direct inhibition hypothesis fails to explain why lesions of the GPe can lead to parkinsonism yet PD can be reversed by GPe DBS [54]. Moreover, the reduction in somatic activity doesn’t necessarily result in the decreased output of the stimulated nuclei. In fact, there are experimental evidences which suggest that the output from the stimulated nucleus(e.g. GPi and STN) is increased due to the activation of axons [29]. In other words, the effects of stimulation on somatic and axonal activity are decoupled [55]. The somatic inhibition maybe caused by depression of excitatory afferents [29]. On the other hand, the axonal excitation results in activation of efferents [28, 29].

**Fig 7:**
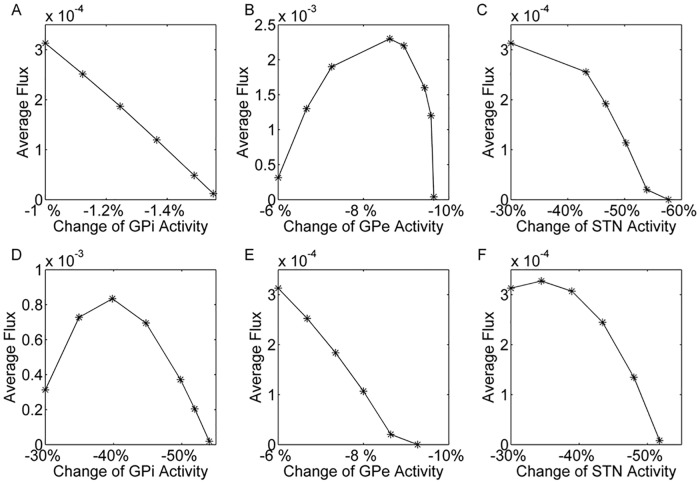
Exploring the mechanism of DBS in terms of average flux. (A-C) show how the flux changes during DBS of GPi, GPe and STN based on the direct inhibition hypothesis. (D-F) show how the flux changes during DBS of GPi, GPe and STN based on the hypothesis that although the DBS inhibits the stimulated target, it may increase the output of the stimulated nucleus.

Here we first try to explore the underlying mechanism of DBS by introducing a negative input directly to the targeting neurons(GPi, GPe, STN, respectively) according to the direct inhibition hypothesis. In this simulation, we considered the neural circuit including the STN nuclei. In the top row of the Fig 7, we show the relationship between the decrease of neural activity in the stimulated target and the average flux that measures the stability and coherence of oscillations in our study. Decrease in the activities of GPi and STN not only reduces the inhibition to thalamic and cortical neurons, but also leads to the monotonic decrease in flux, which means the oscillations become less stable. In the case of stimulations of the GPe, our simulation results show that although the flux may increase first due to the decreased activity in GPe, it also significantly decreases when the GPe is further inhibited. Second, according to the hypothesis of the decoupling effect of DBS, we simulate the inhibitory effect on the stimulated target and the excitatory effect on the corresponding output to the downstream nucleus at the same time. As shown in the bottom row of Fig 7, the flux that drives the oscillations in PD can also be effectively suppressed. Our results point to the validity of both hypotheses on the effect of DBS and suggest that both the abnormal firing rates and firing patterns in the circuit are responsible for the symptoms in PD. Interestingly, according to our theoretical predictions, GPe appears to be another effective stimulated target for DBS besides GPi and STN. Further refinement of the suggested model may lead to the identification of other potential effective target nuclei in DBS for movement disorders.

### Comparison with other models and future directions

About years ago, researchers focused on the altered firing rates along the direct/indirect pathways in basal ganglia when describing the Parkinson’s disease(PD) [9, 13, 14]. With the fact that the presence of synchronized oscillations is a distinctive feature of PD and the symptoms in PD such as tremor cannot be easily explained by the classic firing rate description, recent computational models focused more on the synchronized oscillatory activity in PD.

Many computational models explored the oscillatory behaviors in PD based on the inhibitory-excitatory interactions between the STN and GPe neurons [22, 56–58]. Terman et al. suggested a spiking neuron model of the STN-GPe circuit that the emergence of oscillatory activity results from the increasing inhibitory input from striatum to GPe [56, 57]. However, the oscillations in their model have much lower frequency than the beta range. The lack of beta oscillations may be due to the absence of cortical input. Holgado et al. developed a STN-GPe circuit model where beta oscillations are produced by both the changes in the strength of the interactions between the STN and GPe neurons and the presence of the cortical input [22]. Another model proposed by Leblois et al. suggested that the oscillatory behaviors can be produced by the competition between the direct pathway and the hyper-direct pathway(cortex-STN-GPi) [58]. However, the indirect pathway was ignored in their model.

Although these above mentioned models are capable of producing oscillations, their conclusions are limited by the incomplete structure of the basal ganglia-thalamo-cortical loop. For example, the prediction that the input from cortex to striatum is also very crucial for the emergence of oscillation according to our work and another theoretical study [51] cannot be obtained from the STN-GPe circuit model. In addition, as we have introduced, there are evidences showing that the deep brain stimulation is effective at more than one region of the basal ganglia-thalamo-cortical circuit [28, 29]. Therefore, it is necessary to explore a model including all the main neural modules in the circuit. Our circuit model can provide more details on the corresponding mechanisms in PD and DBS.

In another related work, Frank et al. suggested a model with a similar architecture to ours showing consistent results that the normal action selection function of basal ganglia in motor control is impaired due to dopamine depletion [38]. Different from our current work and other works introduced above that focused on the oscillatory activity in PD, they mainly explored the underlying role of dopamine in learning and execution of cognitive tasks. They showed that both the dopamine depletion in PD and dopamine overdose would impair the learning function [38]. However, the abnormal oscillations in PD are not explicitly explored in their model. Local inhibition or activation is not sufficient to explain all the characteristics in the basal ganglia circuit.

Previous electrophysiological studies have failed to show the expected significant changes of firing rates in the pallidum, thalamus or motor cortical areas of MPTP monkeys [13, 15]. Both abnormal firing rates and firing patterns in the circuit should be paid attentions upon. In our model, the action selection function is affected in PD because the abnormal oscillatory firing behaviors jam normal information processing in the motor circuits. With our landscape and flux theory we are not only able to explore the conditions for the production of oscillations in PD, but also quantitatively explore the stability of oscillatory behaviors through both the topology of the quantified landscape and strength of the flux. Our approach can also help to find effective stimulated targets through quantifying the reduction of the pathological oscillations during DBS in terms of flux.

Frank et al. also showed that the basal ganglia circuit participated in higher level cognitive processes such as decision-making in which two pathways compete with each other and selectively facilitate the execution of the cortical command, which is analogous to the functional roles of basal ganglia in motor control processes. In addition, they have also modeled some behavioral characteristics, such as the speed(reaction time)-accuracy tradeoff in decision-making tasks [59]. In fact, our landscape and flux approach can also help facilitate an understanding of the underlying physical mechanisms of decision-making tasks. In our recently work, we apply our approach to a neural model of the visual motion discrimination task [60]. Differently from previous works, the quantifications of the reaction time and accuracy performance with our method avoided time-consuming calculations from the statistics of the data. We also quantified the energy consumption in the decision-making processes in terms of the entropy production rate. We found that instead of the well-known speed-accuracy tradeoff, there are tradeoffs among decision-speed, accuracy and energy cost in decision-making tasks. In our future works, we’d like to explore the modulatory role of the dopamine/basal ganglia system in decision-making tasks with our landscape and flux theory.

## Conclusion

We explored the dynamics of the basal ganglia-thalamo-cortical circuit from the landscape and flux perspective. The quantified potential landscape is associated with the steady state probability distributions. Dopamine dynamically modulates neural activity in this motor control circuit. Different dopamine levels result in different behaviors. With enough dopamine supply in the basal ganglia circuit, the landscape topography becomes a basin of attraction corresponding to the state with activated motor cortex and thalamus. With dopamine depletion, the system goes through a Hopf bifurcation to beta-band oscillations. The corresponding landscape changes significantly from a monostable basin to a closed oscillation ring. The driving force of the system can be decomposed into the gradient of the potential and the curl flux. The gradient of the potential attracts the system down towards the closed ring, and the flux drives the oscillations along the ring valley.

We used the barrier height and flux to quantify the global stability and coherence of the oscillations in the circuit. Higher barriers accompanied with larger flux lead to more stable and coherent oscillations. Meanwhile, more energies are dissipated. Quantifying the barriers and flux can help us to understand the emergence of abnormal synchronized neuronal activity in movement disorders. Our theoretical predictions quantitatively validate the main hypotheses for the therapeutic mechanism of the deep brain stimulation in the Parkinson’s disease. We find a new potential simulating target for DBS besides the existing GPi and STN. We aim to further refine the suggested model and try to compare our theoretical predictions with clinical data [61, 62].

## Methods and models

### The model of the basal ganglia-thalamo-cortical circuit

As we have discussed in the Introduction section, we consider the whole basal ganglia-thalamo-cortical circuit rather than the inhibitory-excitatory loop architecture of the STN-GPe network for exploring the dynamics in PD [22–24]. A computational model of the modulatory network has been constructed, which includes most of the main modulation modules in the circuit as shown in Fig 1 [8–11, 39–41]. In the present work, we suggest a circuit model consisting of seven main brain regions. They are motor cortex, striatum(direct pathway neurons), striatum(indirect pathway neurons), GPi/SNr complex, GPe, thalamus and STN, which are labeled as 1, 2, 3, 4, 5, 6, 7, respectively in our model. Here, each module in the circuit is represented by a single Hopfield-type model neuron [4]. Similar simplification was also used in previous computational studies [24, 42]. We can write down a set of ordinary differential equations describing the dynamics of this circuit as:

$$\begin{array}{c} F_{1}=\frac{1}{C_{i}}\left(I_{1}-\frac{x_{1}}{R_{1}}+\frac{T_{1,6}*x_{6}^{n}}{s^{n}+x_{6}^{n}}\right) \end{array}$$ (1)

$$\begin{array}{c} F_{2}=\frac{1}{C_{i}}\left(I_{2}-\frac{x_{2}}{R_{2}}+\frac{T_{2,1}*x_{1}^{n}}{s^{n}+x_{1}^{n}}+\frac{T_{2,6}*x_{6}^{n}}{s^{n}+x_{6}^{n}}+D_{input}\right) \end{array}$$ (2)

$$\begin{array}{c} F_{3}=\frac{1}{C_{i}}\left(I_{3}-\frac{x_{3}}{R_{3}}+\frac{T_{3,1}*x_{1}^{n}}{s^{n}+x_{1}^{n}}+\frac{T_{3,6}*x_{6}^{n}}{s^{n}+x_{6}^{n}}-D_{input}\right) \end{array}$$ (3)

$$\begin{array}{c} F_{4}=\frac{1}{C_{i}}\left(I_{4}-\frac{x_{4}}{R_{4}}+\frac{T_{4,7}*x_{7}^{n}}{s^{n}+x_{7}^{n}}-\frac{T_{4,2}*x_{2}^{n}}{s^{n}+x_{2}^{n}}-\frac{T_{4,5}*x_{5}^{n}}{s^{n}+x_{5}^{n}}\right) \end{array}$$ (4)

$$\begin{array}{c} F_{5}=\frac{1}{C_{i}}\left(I_{5}-\frac{x_{5}}{R_{5}}+\frac{T_{5,7}*x_{7}^{n}}{s^{n}+x_{7}^{n}}-\frac{T_{5,3}*x_{3}^{n}}{s^{n}+x_{3}^{n}}\right) \end{array}$$ (5)

$$\begin{array}{c} F_{6}=\frac{1}{C_{i}}\left(I_{6}-\frac{x_{6}}{R_{6}}-\frac{T_{6,4}*x_{4}^{n}}{s^{n}+x_{4}^{n}}\right) \end{array}$$ (6)

$$\begin{array}{c} F_{7}=\frac{1}{C_{i}}\left(I_{7}-\frac{x_{7}}{R_{7}}+\frac{T_{7,1}*x_{1}^{n}}{s^{n}+x_{1}^{n}}-\frac{T_{7,5}*x_{5}^{n}}{s^{n}+x_{5}^{n}}\right) \end{array}$$ (7)

Here, *x*_*i*_ represents the activity of each module in the circuit and *D*_*input*_ is used to represent the dopamine input. The function *f*_*i*_(*x*_*i*_) represents the average firing rate of neural module *i*, which has a monotonic and sigmoid form. In this work we use the Hill function as the form of the response function *f*_*i*_(*x*_*i*_) [34]. *s* and *n* are the coefficients of the Hill function which determine the threshold and steepness of the sigmoidal function. Here we choose the same parameter value that *s* = 2.0 and *n* = 2 as our previous work [34]. *T*_*i*, *j*_ represents the strength of the connection or interaction from neural module *j* to *i*. Here the excitatory connection strengths are set as *T*_1,6_ = *T*_4,7_ = 2, *T*_2,1_ = *T*_2,6_ = *T*_3,1_ = *T*_3,6_ = 1.4, *T*_5,7_ = 1, *T*_1,7_ = 1.8. The inhibitory connection strengths are set as *T*_4,2_ = *T*_5,3_ = *T*_6,4_ = 3.2, *T*_4,5_ = 3.0, *T*_7,5_ = 1.8. We chose these parameter values of the connection strengths according to the previous theoretical modeling work, where the range is 0–7 [51]. *C*_*i*_ and *R*_*i*_ are the membrane capacitance and resistance, respectively. We use *R*_*i*_ = 1.67 according to Hopfield’s previous work [63]. *RC* plays the role of time constant in this model. The realistic time constant *τ* = *RC* is considered as equal to 6*ms* [23]. Therefore, we can have the parameter value of *C*_*i*_ as *C*_*i*_ = *τ*/*R*_*i*_. The external inputs are set as *I*_1_ = 0.1, *I*_2_ = 0.05, *I*_3_ = *I*_7_ = 1.2, *I*_4_ = 4.4, *I*_5_ = 2.8, *I*_6_ = 2. We chose the values of these parameters based on the constrains that the average firing rate of each neural module has to be positive and the steady state solution can switch from mono-stability to beta-band oscillations.

### Self-consistent mean field approximation

In order to obtain the underlying potential landscape that is defined as *U* = −*lnP*_*ss*_, we need to calculate the steady-state probability distribution first. However, due to the huge dimensions of the system, it is often difficult to solve this equation directly. Therefore, we used the self-consistent mean field approximation to reduce the dimensionality [31, 34].

Generally speaking, to obtain the probability *P*(*x*_1_, *x*_2_, …, *x*_*N*_, *t*) of a N-dimensional system, we usually have to solve a N-dimensional partial differential equation. Assuming that every variable has *M* values, then the dimensionality of the system will become *M*^*N*^. This is impossible for numerical calculation when *N* is large. We split the probability into the products of individual ones: $P\left(x_{1},x_{2},...,x_{N},t\right)∼\Pi_{i}^{N}\;P\left(x_{i},t\right)$ with a mean field approach [31, 34]. Then the probability can be solved computationally, since the degrees of freedom are reduced from exponential to polynomials *M* × *N*.

However, it is still often difficult to solve the coupled diffusion equation directly. Fortunately, the moment equations are usually relatively easy to obtain. In principle, if we have the information of all the moments, we can construct the probability distribution. In many cases, not all the moments can be reached. We can start from the moment equations and assume there are specific relations between moments [31, 34]. Here we use Gaussian distribution as an approximation ansatz. Then we need two moments, mean and variance.

In the case of small diffusion coefficient *D*, the moment equations for neural networks can then be approximated as

$$\begin{array}{c} \dot{u(t)}=C\left[u\left(t\right)\right], \end{array}$$ (8)

$$\begin{array}{c} \dot{\sigma (t)}=\sigma \left(t\right)A^{T}\left(t\right)+A\left(t\right)\sigma \left(t\right)+2D\left[x\left(t\right)\right], \end{array}$$ (9)

Here **x** and *σ*(**t**) represent the mean and variance, respectively. The matrix **A** is defined as $A_{ij}=\frac{\partial C_{i}\left[x\left(t\right)\right]}{\partial x_{j}\left(t\right)}$. We consider here only the diagonal element of *σ*(**t**) from the mean field approximation. Then the evolution of probability distribution for each variable can be obtained through the mean and variance with Gaussian approximation:

$$\begin{array}{c} P\left(u,t\right)=\frac{1}{\sqrt{2\pi }\sigma \left(t\right)}exp-\frac{{\left[u-\bar{u}\left(t\right)\right]}^{2}}{2\sigma (t)}, \end{array}$$ (10)

The total probability is equal to the product of probability for each individual variable from the mean field splitting approximation. For mono-stable states, we can construct the potential landscape by: *U* = −*lnP*_*ss*_. However, for the system with oscillations, the mean and variance **x**(**t**) and *σ*(**t**) evolve with time. In fact, the mean represents the deterministic oscillatory dynamics. On the other hand, the variance gives the spread or the width of the fluctuations around the deterministic oscillatory trajectory. Although the deterministic trajectory is oscillatory, the corresponding probability does not change with respect to time. In order to obtain the steady state probability that doesn’t change with time, we quantified the steady state probability distribution by integrating of the probability in time for one period and dividing for that period. Such method is also used in previous works [31, 34].

## Supporting information

S1 Text Supplementary results. (PDF)

S1 Fig Potential landscapes in the 2-dimensional state space of GPi and STN activity for different dopamine levels. (TIF)

S2 Fig The effects of dopamine on the motor circuit in terms of barrier height, flux, period and entropy production rate based on the state space of GPi and STN activity. (TIF)

S3 Fig Potential landscapes of the modulation circuit in the state space of GPi and STN activity for different interaction strength. (A): Increased inhibitory connection in the indirect pathway. (B): Increased inhibitory connection in the indirect pathway.
(TIF)

S4 Fig The global sensitivity analysis in terms of the barrier height and flux based on the state space of GPi and STN activity. (TIF)
