# Supplementary material for: Quantification of motor network dynamics in Parkinson’s disease by means of landscape and flux theory
Source: PLoS One. 2017 Mar 28;12(3):e0174364. doi: 10.1371/journal.pone.0174364 (PMC5370118; doi:10.1371/journal.pone.0174364)
Supplement: S1 Text — (PDF) [file pone.0174364.s001.pdf]

Quantification of motor network dynamics in Parkinson's disease by means of landscape and flux theory

Han Yan[1], Jin Wang[1,2,*]

1 State Key Laboratory of Electroanalytical Chemistry, Changchun Institute of Applied Chemistry, Chinese Academy of Sciences, Changchun, Jilin, P.R.China
2 Department of Chemistry and Physics, State University of New York at Stony Brook, Stony Brook, New York, United States of America

∗ **E-mail: jin.wang.1@stonybrook.edu**

Supplementary Results

The curl flux term J_{ss}/P_{ss} approximately plays the role of velocity when the oscillating system moves in the state space[1, 2]. It is natural to expect that shorter period of oscillations accompanied by larger flux. Our results in Fig.4(B) and (C) of the main text are not incompatible with this prediction. It is because the period is determined by both the flux(velocity) and the loop length of the cycle. In Fig.S1. we can see that for the original circuit without changing and wirings and the circuits with enhanced specific connection the flux multiplied by period has positive correlations with loop length.

In the main text, we show the results based on cortical and thalamic activity. To address whether the results are also valid for the other nuclei activities, here we also show corresponding results in the state space of GPi and STN activity in the following figures. We can see that choosing any two nuclei activities for the 2-dimensional state space does not significantly change the theoretical predictions we get.

Entropy production rate

For non-equilibrium neural network dynamics, the driving force can be decomposed into a gradient of the potential and a curl flux force as:$\mathbf{F} = -\mathbf{D}\nabla U + \mathbf{J_{SS}}/\mathbf{P_{SS}}$. And the entropy production rate can be quantified as:$epr = \int dI(\mathbf{J}\cdot\mathbf{D^{-1}}\cdot\mathbf{J})/P$.[2]

References

[1] Yan H, Zhao L, Hu L, Wang XD, Wang EK, Wang J. Nonequilibrium landscape theory of neural networks. Proceedings of the National Academy of Sciences of the United States of America. 2013;110(45):E4185–E4194.

[2] Zhang F, Xu L, Zhang K, Wang EK, and Wang J. The potential and flux landscape theory of evolution. The Journal of Chemical Physics. 2012;137(6):2840–2847.

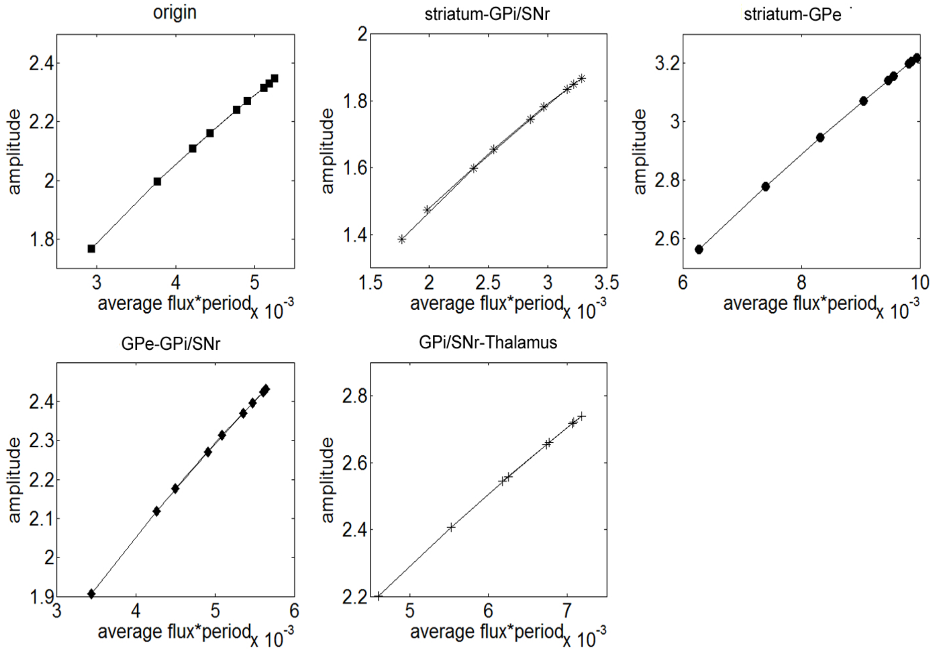

Figure 1: In both original circuit and circuits with enhanced specific connection, the flux multiplied by period has positive correlations with loop length.
