# Supplementary figures and images for: Quantification of motor network dynamics in Parkinson’s disease by means of landscape and flux theory
Source: PLoS One. 2017 Mar 28;12(3):e0174364. doi: 10.1371/journal.pone.0174364 (PMC5370118; doi:10.1371/journal.pone.0174364)

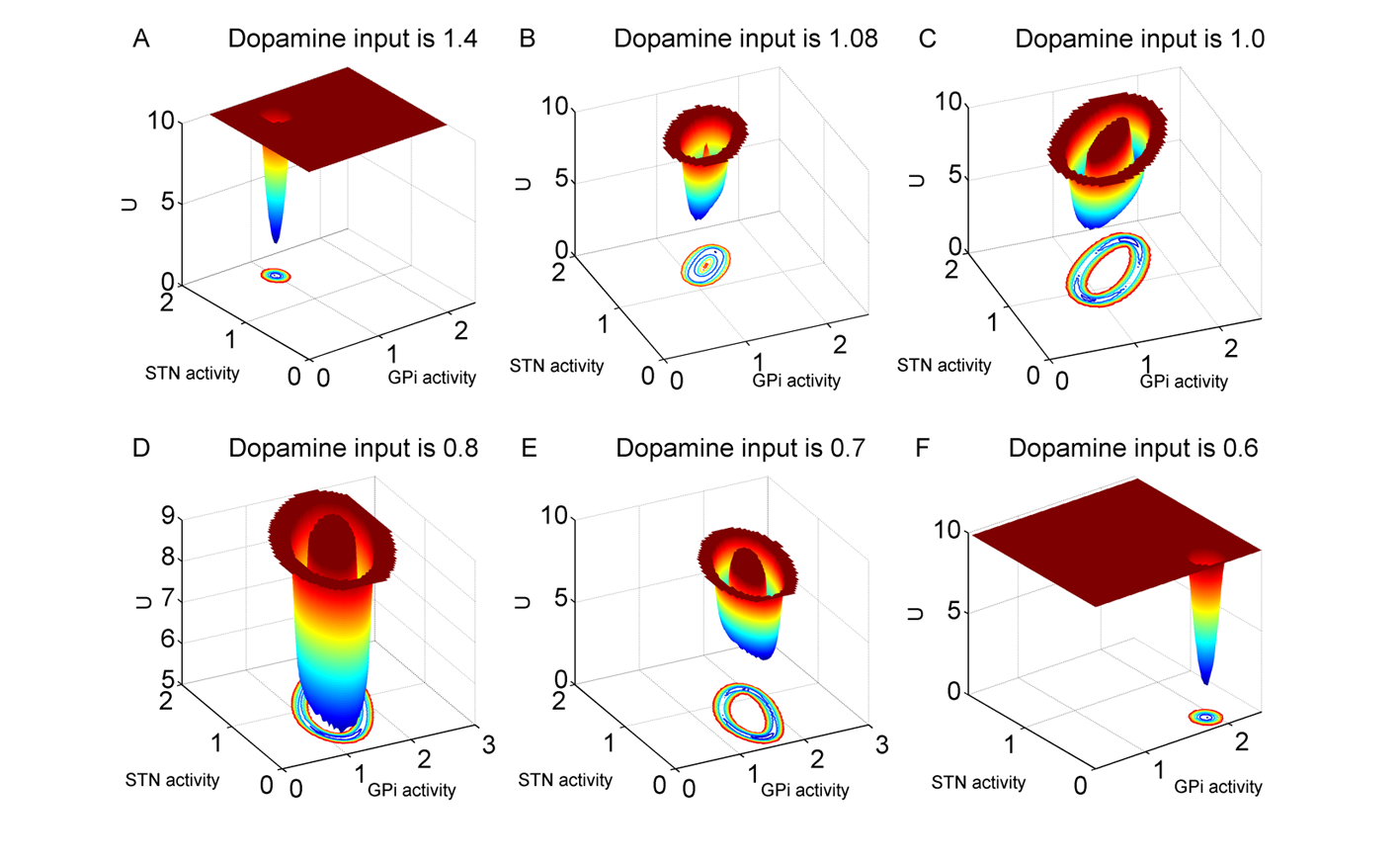

Supplement: S1 Fig — (TIF) [file pone.0174364.s002.tif]

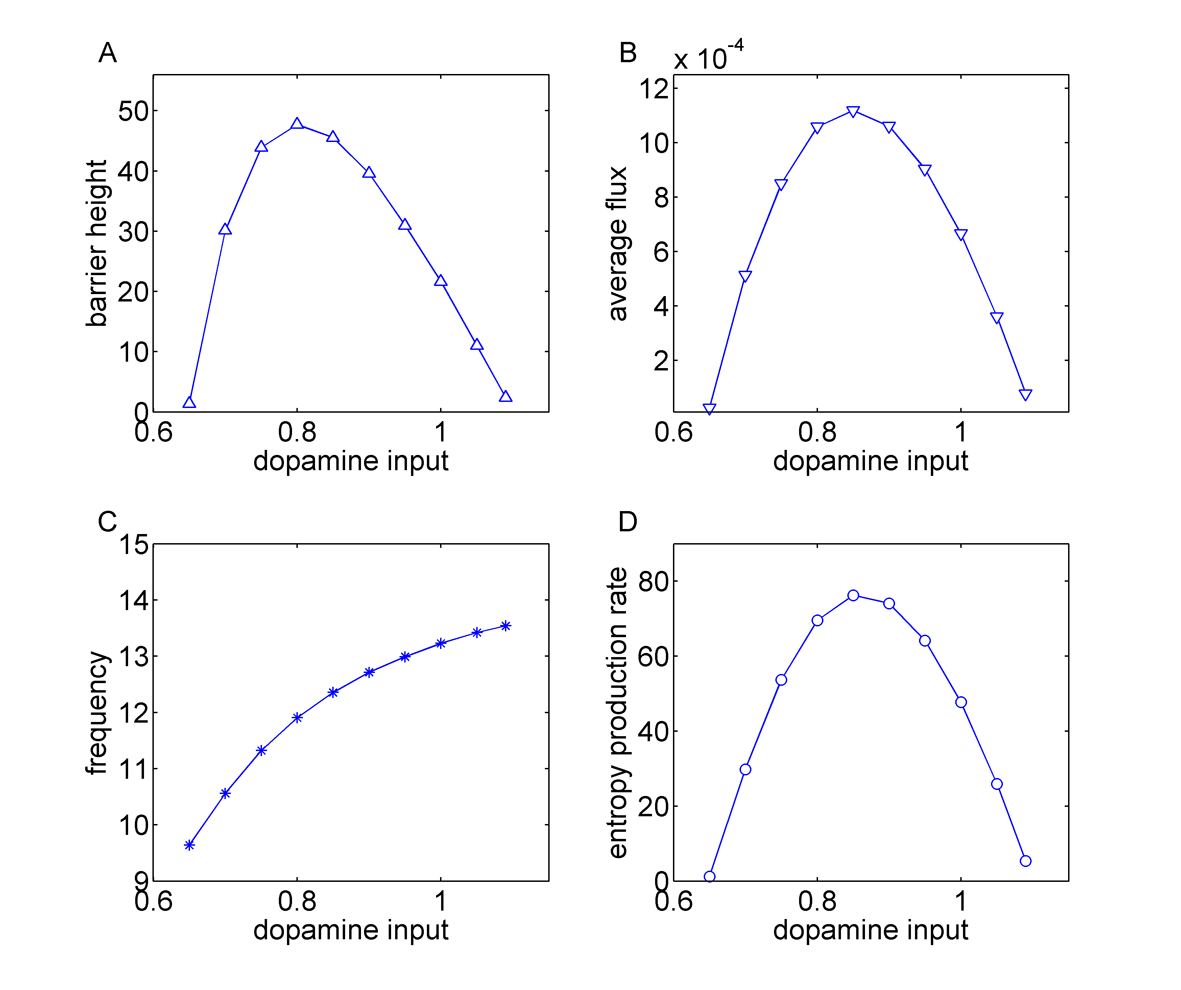

Supplement: S2 Fig — (TIF) [file pone.0174364.s003.tif]

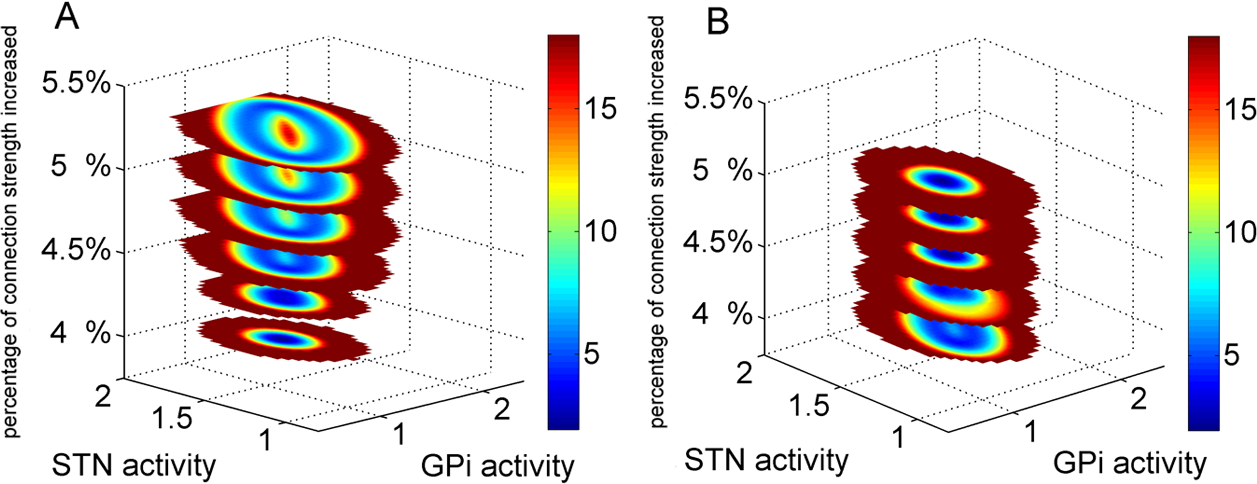

Supplement: S3 Fig — (A): Increased inhibitory connection in the indirect pathway. (B): Increased inhibitory connection in the indirect pathway. (TIF) [file pone.0174364.s004.tif]

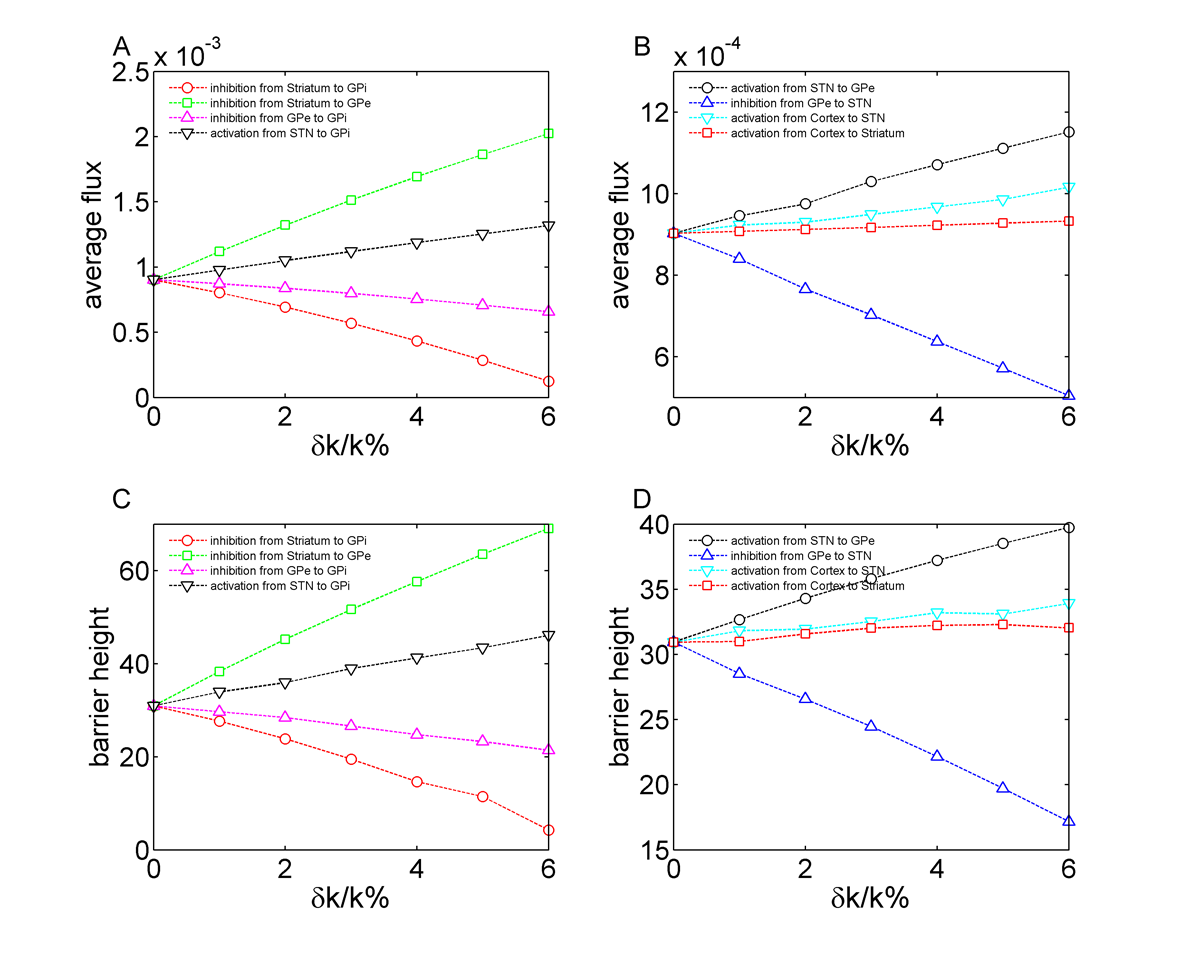

Supplement: S4 Fig — (TIF) [file pone.0174364.s005.tif]
